# Case of Transfusion

**Published:** 1873-12

**Authors:** 


Case of Transfusion.—*Under the care of Mr. J. McCarthy.* His condition was so bad that I hesitated as to meddling with him, he seemed so nearly dead; but as blood was trickling from the wound, I had him removed to the theatre, and without any chloroform explored the wound. I found very deep down a large vessel with a gaping wound in the side of it. I secured this with double ligature and all bleeding stopped. The man was now pulseless and scarcely breathing. I determined to try transfusion. Mr. Rudduck, one of the dressers of the week, at once volunteered, but Mr. Latham, the other dresser to whom the case would belong, claimed the preference, and supplied the required blood.

While the house surgeon bled Mr. Latham, I exposed the right medio-cephalic vein and passed a director under it. The blood was quickly whipped and twice strained through some fine linen into a tumbler placed in a basin of warm water. The injecting apparatus was a syringe on the principle of the stomach-pump syringe. All being now ready, air having been completely got rid of, I made a small opening with a pair of scissors into the exposed vein, and forcing a small stream of blood from the muzzle, so passed it into the vein. Injecting slowly, about twenty drachms, I observed that the respiration at once improved, becoming full and regular; but after about eighteen such respirations, the interval between each became longer, and I stopped the injection. He then rapidly sank; and, notwithstanding artificial respiration, was dead in about ten minutes.

